# Gypenosides ameliorate high-fat diet-induced non-alcoholic steatohepatitis *via* farnesoid X receptor activation

**DOI:** 10.3389/fnut.2022.914079

**Published:** 2022-08-24

**Authors:** Hongshan Li, Yingfei Xi, Hongliang Liu, Xin Xin

**Affiliations:** ^1^Liver Disease Department of Integrative Medicine, Hwa Mei Hospital, University of Chinese Academy of Sciences, Ningbo, China; ^2^Key Laboratory of Diagnosis and Treatment of Digestive System Tumors of Zhejiang Province, Ningbo, China; ^3^Shuguang Hospital, Institute of Liver Disease, Shanghai University of Traditional Chinese Medicine, Shanghai, China

**Keywords:** farnesoid X receptor, gypenosides, high-fat diet, non-alcoholic steatohepatitis, mice

## Abstract

**Background:**

Gypenosides (Gyps), the major botanical component of *Gynostemma pentaphyllum*, was found to up-regulate the farnesoid X receptor (FXR) in a mouse model of non-alcoholic steatohepatitis (NASH). However, the exact role of FXR and underlying mechanisms in Gyps-mediated effects on NASH remain to be elucidated.

**Purpose:**

This study investigated whether Gyps attenuates NASH through directly activating FXR in high-fat diet (HFD)-induced NASH, and delineated the molecular pathways involved.

**Study design:**

A mouse model of HFD-induced NSAH was used to examine effects of Gyps on NASH with obeticholic acid (OCA) as a positive control, and the role of FXR in its mechanism of action was investigated in wild-type (WT) and FXR knockout (KO) mice.

**Methods:**

WT or FXR KO mice were randomly assigned into four groups: normal diet (ND) group as negative control, HFD group, HFD + Gyps group, or HFD + OCA group.

**Results:**

Treatment with Gyps and OCA significantly improved liver histopathological abnormalities in HFD-induced NASH, reduced the non-alcoholic fatty liver disease (NAFLD) activity score (NAS), and lowered hepatic triglyceride (TG) content compared with the HFD group. In agreement with these liver tissue changes, biochemical tests of blood samples revealed that alanine aminotransferase (ALT), aspartate aminotransferase (AST), TG, total cholesterol (TC), low-density lipoprotein cholesterol (LDL-C), fasting blood glucose (FBG), and fasting insulin (FINS) levels were significantly lower in the HFD + Gyps vs. HFD group. Furthermore, Gyps and OCA treatment significantly up-regulated hepatic FXR, small heterodimer partner (SHP), carnitine palmitoyltransferase 1A (CPT1A), and lipoprotein lipase (LPL) expression, and significantly down-regulated sterol-regulatory element binding protein 1 (SREBP1), fatty acid synthetase (FASN), and stearoyl-CoA desaturase 1 (SCD1) protein levels compared with the HFD group in WT mice but not in FXR KO mice. Notably, Gyps- and OCA-mediated pharmacological effects were significantly abrogated by depletion of the FXR gene in FXR KO mice.

**Conclusion:**

Gyps ameliorated HFD-induced NASH through the direct activation of FXR and FXR-dependent signaling pathways.

## Introduction

Non-alcoholic steatohepatitis (NASH) is a severe form of non-alcoholic fatty liver disease (NAFLD) that is characterized by hepatic injury and inflammation due to excessive accumulation of fat in the liver. NASH can progress to liver cirrhosis and even hepatocellular carcinoma (HCC), which can cause serious outcomes and may require liver transplantation (1). As a liver manifestation of obesity-related metabolic syndrome, NAFLD includes simple steatosis and NASH, and if the disease progresses, liver cirrhosis and HCC (2). NAFLD is highly prevalent, affecting approximately 25% of the population worldwide, with a prevalence of 23.71% in Europe, and 27.37% in Asia (3). It has been noted that the incidence of NAFLD and its clinically aggressive form, NASH, are on the rise, and that they pose serious economic and health burdens (3). More importantly, NASH can induce cardiovascular events and HCC, which highlights the urgency of clinical treatment. Currently, lifestyle-based interventions including dietary modifications and weight loss *via* exercise are considered the primary means for treatment of this disease (4). However, to date no pharmacological therapies for NASH have been approved.

*Gynostemma pentaphyllum* (Thunb.) Makino is an herbaceous climbing plant in the Cucurbitaceae family. As a safe and accessible natural herb, it has long been used to treat various chronic diseases in China and other Asian countries (5) due to its multiple pharmacological effects including antifibrotic, anti-inflammatory, antioxidant, and anticancer properties (6–9). Gypenosides (Gyps) are the main active components of *G. pentaphyllum* and have been shown to inhibit fat deposition and ameliorate fibrosis in the liver (10, 11). Most recently, we found that Gyps exerted a therapeutic effect during NASH (12, 13), in which the farnesoid X receptor (FXR) and its signaling pathway were up-regulated in a mouse model of high-fat diet (HFD)-induced NASH (12). However, whether the Gyps-mediated beneficial effects could be achieved independent of FXR and its signaling pathway remains unclear.

Intrigued by and building upon our previous research, the current study was performed using FXR knockout (KO) mice and aimed to gain further insight into the mechanism underlying the effect of Gyps on NASH, as well as to clarify the role of FXR in this mechanism. The resulting data will provide a better understanding of the mechanisms underlying the therapeutic effects of Gyps on NASH and may lay the foundation for a novel treatment for patients with NASH.

## Materials and methods

### Gyps and ingredient analysis

Gyps (batch number, 180527; specification > 98%) was purchased from Shanghai Winherb Medical Technology Co., Ltd. (Shanghai, China). Gyps (27.2 mg) was fully dissolved in methanol (2 mL), then the solution was filtered. The fingerprint spectrum was analyzed using a Dionex Ultimate 3,000 ultrahigh-performance liquid chromatography system (Thermo Scientific, United States) and a Thermo Scientific Q Exactive quadrupole-electrostatic field orbitrap high-resolution mass spectrometry system. Detailed information regarding these analyses was previously described (14).

**FIGURE 1:**
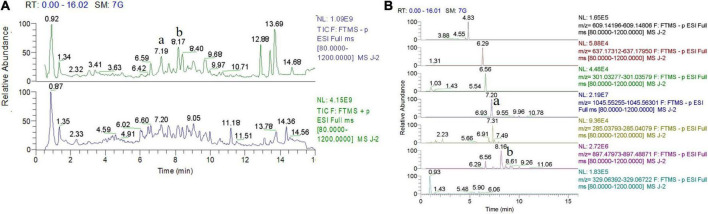
Total-ion chromatogram and negative-ion chromatogram for gypenosides (Gyps). **(A)** Total-ion chromatogram for Gyps. **(B)** Negative-ion chromatogram for Gyps. a: gypenoside A; b: gypenoside XLIX. The total-ion chromatogram and negative-ion chromatogram for the corresponding standards were provided as we previously reported (12).

High-performance liquid chromatography and mass spectrometry analysis revealed that gypenoside A (molecular formula: C_46_H_74_O_17_) and gypenoside XLIX (molecular formula: C_52_H_86_O_21_) were the main components of Gyps (220.61 mg/g Gyps and 344.10 mg/g Gyps, respectively). The total-ion chromatogram and negative-ion chromatogram for Gyps are shown in Figure 1. During the analysis, the corresponding standards were used as reported in our previous study (12).

OCA (batch no. 3j28b18980) was purchased from BioVision Inc. (San Francisco, CA, United States). OCA was prepared and administrated as described previously (12).

### Experimental animals and study design

Twenty-three male specific pathogen-free (SPF) C57BL/6 FXR knockout (KO, *FXR*^–/–^) mice (16–20 g) were purchased from Shanghai Model Organisms Centre Inc. [license number: SCXK (Shanghai) 2014-0002]. Breeding mice originated from Jackson Laboratories (United States), and Shanghai Model Organisms Centre Inc. (Shanghai, China). Thirty-six SPF wild-type (WT) C57BL/6 male mice of the same age (16–20 g) were purchased from Shanghai Slake Laboratory Animal Co., Ltd., [license number: SCXK (Shanghai) 2017-0003]. The WT and FXR KO mice were maintained in an animal room at the Shanghai University of Traditional Chinese Medicine (Shanghai, China).

The WT or FXR KO mice were randomly divided into the following groups using the random number table method: (1) normal diet (ND) group (*n* = 9 WT mice; *n* = 6 FXR KO mice; *n* = 5 OCA-treated mice), HFD group (*n* = 9 WT mice; *n* = 6 FXR KO mice), HFD + Gyps group (*n* = 9 WT mice; *n* = 6 FXR KO mice), and HFD + OCA group (*n* = 9 WT mice; *n* = 5 FXR KO mice). The WT or FXR KO mice were given standard chow (cat. no. D12450B; 10% of energy from fat; calories, 3.84 kcal/g; purchased from Research Diets, United States) in the ND group, a high-fat chow (cat. no. D12492i; 60% of energy from fat; calories, 5.21 kcal/g; purchased from Research Diets, United States) in the HFD group, and high-fat chow plus Gyps in the HFD + Gyps group as was previously described (12). The WT or FXR KO mice were initially fed a HFD for 10 weeks and subsequently administered Gyps (100 mg⋅kg^–1^⋅d^–1^ by gavage) or OCA (10 mg⋅kg^–1^⋅d^–1^ by gavage) for an additional 4 weeks. The WT or FXR KO mice in the ND and HFD groups were given an equal volume of drinking water by gavage for the additional 4 weeks.

Upon completion of the 14-week treatments in each group, mice were anesthetized with injections of 3% sodium pentobarbital at 3 mL/kg after fasting for 12 h. Blood samples were collected from the inferior vena cava. Liver tissues were harvested and a small piece of tissue (approximately 0.5 × 0.5 × 0.3 cm) was fixed in 10% neutral buffered formalin for routine hematoxylin and eosin (H&E) staining. The remaining liver tissues were stored in a –70°C ultra-low temperature freezer for western blot (WB) analysis.

The study involving experimental animals was reviewed and approved by the Experimental Animal Ethics Committee of Shanghai University of Traditional Chinese Medicine (Approval No: PZSHUTCM190308010, Approval date: September 21, 2018).

### Histopathological examinations of liver tissues

Liver tissues were fixed in 10% neutral buffered formalin. After dehydration and embedding in paraffin, liver tissues were sectioned (4 μm). Liver sections were subjected to conventional H&E staining and assessed by a liver pathologist under an inverted fluorescence microscope (Reka 37XB, Germany). Based on the histopathological examinations, the NAFLD activity score (NAS) was determined. The NAS included steatosis, inflammation, and ballooning degeneration, and specific criteria were based on previously published research (15): steatosis (< 5%, 0 points; 5–33%, 1 point; 33–66%, 2 points; and > 66%, 3 points), intralobular inflammation (none, 0 points; less than two foci/200 × field, 1 point; two to four foci/200 × field, 2 points; and more than four foci/200 × field, 3 points), and balloon-like changes (not seen, 0 points; rare, 1 point; and more common, 2 points).

### Measurement of hepatic triglyceride content

Hepatic triglyceride (TG) content was determined as described previously (12). In brief, liver samples were homogenized and centrifuged, after which the supernatant was collected for measurement of hepatic TG content using a TG kit from Nanjing Jiancheng Bioengineering Institute (cat. no. F001-1-1; Jiangsu, China) in accordance with the manufacturer’s instructions.

### Biochemical tests

Liver aminotransferases, including alanine aminotransferase (ALT) and aspartate aminotransferase (AST) activities, were determined using ALT (cat. no. C009-2-1) and AST (cat. no. C010-2-1) kits (Nanjing Jiancheng Bioengineering Institute; Jiangsu, China) following the manufacturer’s instructions as described in our previous study (12).

Lipid profiles, including serum TG, total cholesterol (TC), low-density lipoprotein cholesterol (LDL-C), and high-density lipoprotein cholesterol (HDL-C), were measured with the following kits: TG (cat. number: F001-1-1), TC (cat. number: A111-1-1), LDL-C (cat. number: A113-1-1), and HDL-C (cat. number: A112-1-1; Nanjing Jiancheng Bioengineering Institute) according to the manufacturer’s instructions as described in our previous study (12).

Fasting blood glucose (FBG) was measured using the glucose oxidase method following the instructions provided with the kit (cat. no. F006-1-1; Nanjing Jiancheng Bioengineering Institute). Serum fasting insulin (FINS) levels were measured using an enzyme-linked immunosorbent assay (ELISA) kit. The mouse insulin kit was purchased from Crystal Chem Inc. (cat. no. 90080; Downers Grove, United States) and FINS levels were determined following the protocol provided by the manufacturer as described in our previous study (12).

The insulin concentrations were calculated using a standard curve, and an additional variable was determined with the following equation: homeostasis model assessment insulin resistance (HOMA-IR) = FBG × FINS/22.5.

### Quantitative RT-polymerase chain reaction

Total RNA was extracted from liver tissue using the RNA extraction kit (Shenggong Bioengineering (Shanghai) Co., Ltd., batch No.: e928ka9723) following the manufacturer’s instructions as described in our previous study (12). RNA concentration was measured using a TECAN Infinite 200 Pro (Tecan Group Ltd., Switzerland) at 260 nm. Reverse transcription (RT) was performed using iScript™ cDNA Synthesis Kit (Bio-Rad, Hercules, United States; cat. no. 170–8891). Primer sequences for RT-PCR analysis were described in our previous study (12). qPCR was performed with TB Green™ Premix Ex Taq™ (TaKaRa, Japan, cat. no. RR420A) and a QuantStudio™ Real-time PCR system (Applied Biosystem, Foster City, United States). Relative mRNA expression of an interest gene was normalized to that of β-actin, a widely used internal reference gene for RT-PCR analysis.

### Western blot analysis

WB analysis was conducted to examine the expression of proteins of interest. Specific primary antibodies used for WB analysis included: farnesoid X receptor (FXR; cat. no. A9033A, Thermo Fisher; 1:1,000 dilution); small heterodimer partner (SHP; cat. no. PA5-76632, Thermo Fisher; 1:1,000); sterol-regulatory element binding protein 1 (SREBP1; cat. no. ab28481, Abcam; 1:1,000); stearoyl-CoA desaturase 1 (SCD1; cat. no. ab19862, Abcam; 1:1,000); carnitine palmitoyltransferase 1A (CPT1; cat. no. ab128568, Abcam; 1:1,000); lipoprotein lipase (LPL; cat. no. ab21356, Abcam; 1:1,000); fatty acid synthetase (FASN; cat. no. 3189, CST; 1:1,000); glyceraldehyde-3-phosphate dehydrogenase (GAPDH; cat. no. 10494-1-AP, Proteintech Group Inc.; 1:10,000); and β-actin (cat. no. 20536-1-AP, Proteintech Group Inc.; 1:5,000).

For WB analysis, total proteins were isolated from liver tissues using radioimmunoprecipitation assay (RIPA) lysis buffer (Beyotime, cat. no. P0013B) with protease inhibitors (Beyotime, cat. no. P1045-1) and phosphatase inhibitors (Beyotime, cat. no. P1045-2). Protein concentration was quantified using the Pierce™ BCA Protein assay kit (Thermo Fisher, cat. no. TI269557) according to the manufacturer’s instructions. The protein samples were loaded onto a gel for sodium dodecyl sulfate–polyacrylamide gel electrophoresis (SDS-PAGE). Subsequently, the proteins were transferred onto polyvinylidene fluoride (PVDF) membranes for protein detection as described previously (12). In brief, after blocking, the diluted primary antibody was added and membranes were incubated overnight at 4°C, followed by incubation with the secondary antibody (goat anti-mouse IgG or goat anti-rabbit IgG; Beijing Dingguo Changsheng Biotechnology Co., Ltd., cat. no. IH-0031 or IH-0011, respectively) the following day at room temperature (RT) for 1 h. After washing with tris-buffered saline with Tween20 (TBST), enhanced chemiluminescence (ECL) developing solution (BeyoECL Plus solution A and solution B; Beyotime, cat. no: P0018 M-1 and P0018 M-2, respectively) was used to visualize the protein bands. Images were developed using chemical imaging analysis software (ChemiAnalysis; Clinx Science Instrument Co., Ltd., Shanghai, China), and the gray value of each band was analyzed using image analysis software (ChemiScope 3000 mini; Clinx Science Instrument Co., Ltd., Shanghai, China). Detailed methods for WB analysis were described in our previous study (12).

### Statistical analysis

SPSS version 21.0 statistical software was used for statistical analysis. The data were tested for normality and homogeneity of variance. Normally distributed data were shown as mean ± standard deviation (SD), and non-normally distributed data were presented as the median (min-max). For data with a normal distribution and homogeneous variance, one-way analysis of variance (ANOVA) was carried out to evaluate the differences among multiple groups, and the least-significant difference (LSD) test was performed to distinguish the differences between two groups. For data without normal distribution or with non-homogeneous variance, the Kruskal-Wallis test was conducted to compare differences among multiple groups, and the Games-Howell test was performed to distinguish the differences between two groups. Results were considered statistically significant when the test level was α = 0.05 and *p* < 0.05.

## Results

### Gyps attenuated high-fat diet-induced body weight gain in wild-type but not farnesoid X receptor knockout mice

**FIGURE 2:**
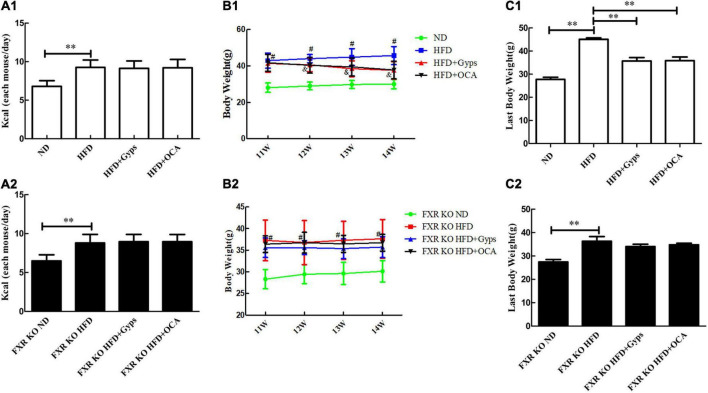
Effects of Gyps on food intake and body weight of WT and FXR KO mice in different groups. **(A1,A2)** Changes in food intake in WT and FXR KO mice in different groups. **(B1,B2)** Changes in body weight in WT and FXR KO mice at different time points (11, 12, 13, and 14 weeks) after treatment in WT and FXR KO mice; ^#^*p* < 0.01, vs. ND group; ^&^*p* < 0.01, vs. HFD group. **(C1,C2)** Changes in body weight in WT and FXR KO mice at the end of experiments in WT and FXR KO mice. WT, wide-type; KO, knockout; ND, normal diet; HFD, high-fat diet; Gyps, gypenosides. ^**^p < 0.01.

Initially, the effects of Gyps and OCA on food intake and body weight of WT and FXR KO mice in the different experimental groups, including ND, HFD, HFD + Gyps and HFD + OCA, were examined. As illustrated in Figures 2A1,A2, food intake was considerably higher in the HFD group vs. the ND group in both WT and FXR KO mice (*p* < 0.01), whereas there was no significant difference in food intake among the HFD + Gyps, HFD + OCA, and HFD groups in both WT and FXR KO mice (*p* > 0.05).

There was significant difference in body weight of WT mice in the HFD + Gyps and HFD + OCA group compared with the HFD groups from the 12th week (*p* < 0.01), but no such significant difference was observed in FXR KO mice (Figures 2B1,B2). Upon completion of the experiments, the body weight gained in WT and FXR KO mice was significantly higher in the HFD group vs. the ND group (all *p* < 0.01). However, the body weight gained was significantly lower in the HFD + Gyps and HFD + OCA groups compared with the HFD group in WT mice (*p* < 0.01), indicating that Gyps or OCA significantly attenuated HFD-induced body weight gain, but this effect of Gyps or OCA was not found in FXR KO mice (*p* > 0.05; Figures 2C1,C2). There was no significant difference in body weight between HFD + Gyps and HFD + OCA groups.

The general conditions of the experimental mice were monitored daily, and the mice in the ND group presented with normal activity, while the mice in the HFD group showed decreased activity. The general conditions of the mice in the HFD + Gyps or HFD + OCA groups were more similar to those in the ND group.

### Gyps improved hepatic steatosis in wild-type but not farnesoid X receptor knockoutmice

**FIGURE 3:**
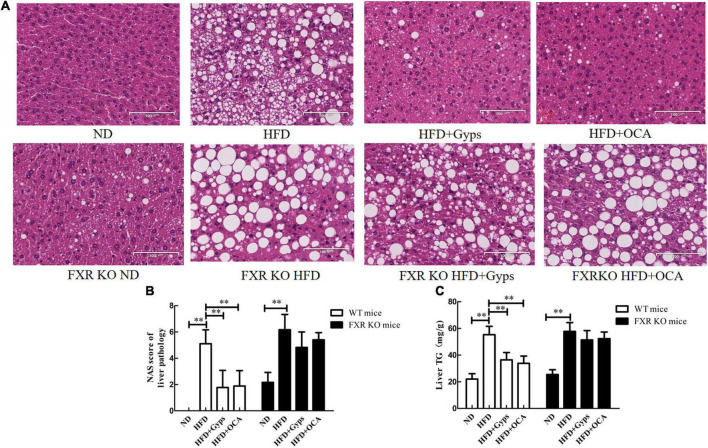
Effects of Gyps on histopathological alterations of liver sections and hepatic TG levels in WT and FXR KO mice. **(A)** Representative images of liver histopathological alterations in different groups. H&E staining of liver sections of WT mice (upper panels) and FXR KO mice (lower panels) in the ND, HFD, and HFD + Gyps groups. The characteristic histological findings of hepatic steatosis were observed in the HFD group, including many fat droplets in the cytoplasm, scattered inflammatory cell infiltration, and balloon-like degeneration. **(B)** Comparison of activity grade between groups in WT and FXR KO mice according to NAS score of liver pathology. **(C)** Levels of hepatic TG in liver tissue of mice in each group. Comparison of hepatic TG levels between groups in WT and FXR KO mice. WT, wild-type C57BL/6 mice (*n* = 9); *FXR*^–/–^, FXR knockout (KO) C57BL/6 mice (*n* = 6); FXR, farnesoid X receptor; TG, triglyceride; ND, normal diet; HFD, high-fat diet; Gyps, gypenosides; NAS, non-alcoholic fatty liver disease (NAFLD) activity score ^**^p < 0.01.

Histopathological examinations of liver sections revealed characteristic changes of hepatic steatosis, including deposition of many fat droplets in the cytoplasm, scatted inflammatory cell infiltration, and balloon-like degeneration in the HFD group, whereas normal histology was observed in the ND group (Figure 3A). These histopathological findings demonstrated that a mouse model of HFD-induced NASH was successfully established. Interestingly, the WT mice in the HFD + Gyps or HFD + OCA groups exhibited an obvious improvement in HFD-induced hepatic steatosis with fewer fat droplets and decreased hepatic inflammation compared to the mice in the HFD group, indicating that treatment with Gyps or OCA attenuated HFD-induced NASH (upper panels, Figure 3A).

Furthermore, the histopathological findings between groups of FXR KO mice were compared. As shown in the lower panels of Figure 3A, the liver structure was mostly normal in the ND group with slightly scattered hepatocyte steatosis, whereas the HFD group showed obvious hepatic steatosis with a large amounts of fat droplets in the cytoplasm, scattered inflammatory cell infiltration, and balloon-like degeneration. It was noteworthy that the hepatic steatosis was not improved following Gyps or OCA treatment, and similar levels were observed among the HFD + Gyps, HFD + OCA, and HFD groups (lower panels, Figure 3A).

Compared with the liver tissue of mice in the ND group, the NAS scores and TG levels in the liver tissue of mice in the HFD group were significantly increased (*p* < 0.01; Figures 3B,C). In agreement with the histopathological findings, the NAS scores and TG levels in the liver tissue of WT mice in the HFD + Gyps or HFD + OCA groups were significantly decreased compared to the HFD group (*p* < 0.01). There was no significant difference in NAS scores and TG levels in the liver tissue of mice between the HFD + Gyps and HFD + OCA groups (*p* > 0.05).

Compared with mice in the FXR KO ND group, the NAS scores and TG levels in the liver tissue of mice in the FXR KO HFD group were significantly increased (*p* < 0.01), and compared to the FXR KO HFD group, there were no significant changes in NAS scores or TG levels in the liver tissue of mice in the FXR KO HFD + Gyps group or FXR KO HFD + OCA group (*p* > 0.05; Figures 3B,C).

### Gyps significantly decreased serum alanine aminotransferase and aspartate aminotransferase activities, lipid levels, and blood sugar parameters in wild-type but not farnesoid X receptor knockout mice

**FIGURE 4:**
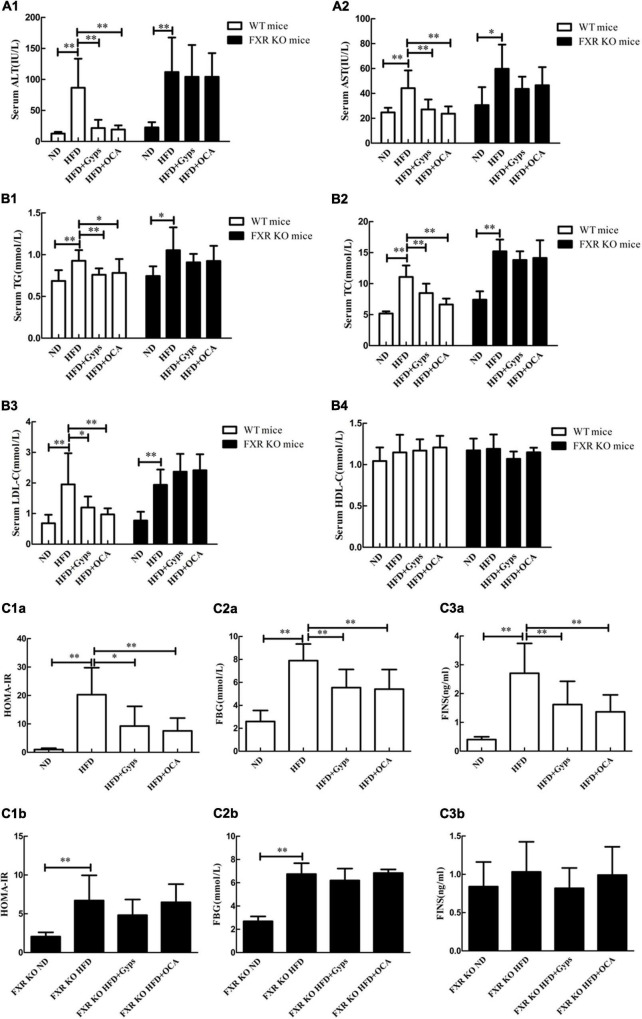
Effects of Gyps on biochemical parameters in WT and FXR KO mice. Alterations in serum levels of **(A1,A2)** liver transaminases (ALT, AST), **(B1–B4)** lipid profiles (TG, TC, LDL-C, HDL-C), and **(C1a–C3b)** blood sugar levels (FINS, FBG, HOMA-IR) in the ND, HFD, or HFD + Gyps groups in WT and FXR KO mice. WT, wild-type C57BL/6 mice (*n* = 9); KO, FXR knockout C57BL/6 mice (*n* = 6); TG, triglyceride; FXR, farnesoid X receptor; ALT, alanine aminotransferase; AST, aspartate aminotransferase; TC, total cholesterol; LDL-C, low-density lipoprotein cholesterol; HDL-C, high-density lipoprotein cholesterol; ND, normal diet; HFD, high-fat diet; Gyps, gypenosides; FINS, fasting insulin; FBG, fasting blood glucose; HOMA-IR, homeostasis model assessment insulin resistance *p < 0.05, ^**^p < 0.01.

In WT mice, serum ALT and AST activities as well as lipid levels (TG, TC, LDL-C) were significantly higher in the HFD group compared to the ND group (*p* < 0.01; Figures 4A1,A2,B1–B4). Compared with the HFD group, the serum ALT and AST activities, and levels of TG, TC, and LDL-C in HFD + Gyps or HFD + OCA groups were significantly increased (*p* < 0.01 or *p* < 0.05; Figures 4A1,A2,B1–B4). There was no significant difference in serum HDL-C among all experimental groups (*p* > 0.05).

In FXR KO mice, the HFD group had significantly higher serum ALT and AST activities, as well as lipid levels (TG, TC, and LDL-C), in comparison with the ND group (*p* < 0.01 or *p* < 0.05; Figure 4). In addition, serum TC and LDL-C levels were significantly higher in HFD FXR KO mice than those of HFD WT mice (*p* < 0.01; Figure 4). Notably, there were no significant differences in serum ALT and AST activities or lipid levels (TG, TC and LDL-C) between the HFD + Gyps and HFD groups (*p* > 0.05; Figure 4). These findings suggested that Gyps significantly decreased serum ALT and AST activities, as well as lipid levels (TG, TC, and LDL-C) in WT but not FXR KO mice.

In WT mice, the serum FINS and FBG levels, and HOMA-IR were significantly increased in the HFD group vs. ND group (*p* < 0.01), while Gyps treatment significantly decreased the serum FINS and FBG levels, and HOMA-IR in the HFD + Gyps group vs. the HFD group (*p* < 0.01 or *p* < 0.05; Figures 4C1a–3b). There was no significant difference in serum FINS and FBG levels, or HOMA-IR between HFD + Gyps and HFD + OCA groups. In FXR KO mice, the HFD group had significantly higher serum FBG levels and HOMA-IR than those in the ND group (*p* < 0.01), and there were no differences in serum FINS and FBG levels, or HOMA-IR between the HFD + Gyps group and the HFD group (*p* > 0.05; Figures 4C1a–3b). These data indicated that the serum FINS and FBG levels and HOMA-IR were significantly decreased in response to Gyps treatment in WT but not FXR KO mice.

### Gyps up-regulated hepatic mRNA and protein expression levels of farnesoid X receptor and its target small heterodimer partner in a mouse model of high-fat diet-induced non-alcoholic steatohepatitis

**FIGURE 5:**
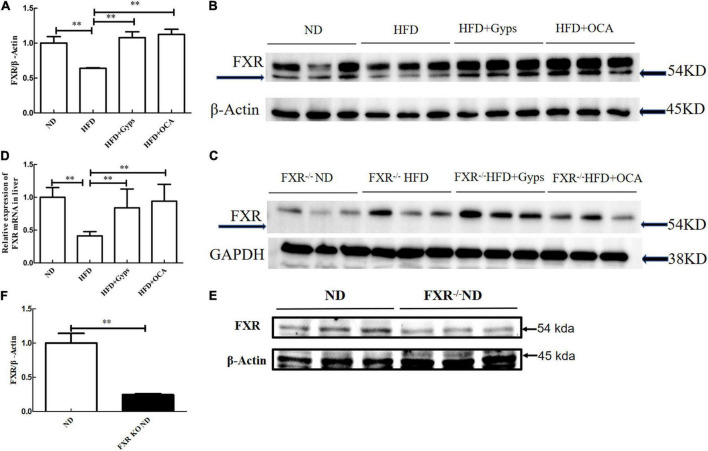
Effects of Gyps on hepatic FXR mRNA and protein expression levels in WT and FXR KO mice. **(A)** Quantification of hepatic FXR protein levels in different groups in WT mice. **(B)** WB analysis of hepatic FXR and β-actin protein expression in WT mice. **(C)** WB analysis of hepatic FXR and GAPDH protein expression in FXR KO mice. **(D)** Relative expression levels of FXR mRNA in different groups in WT mice. **(E)** WB analysis of hepatic FXR protein expression in the ND and FXR KO ND groups. **(F)** Quantification of hepatic FXR protein levels in the ND and FXR KO ND groups. FXR, farnesoid X receptor; GAPDH, glyceraldehyde 3-phosphate dehydrogenase; ND, normal diet; HFD, high-fat diet; Gyps, gypenosides; WT, wild-type; KO, knockout; WB, western blot ^**^p < 0.01.

Next, RT-PCR and WB analyses were performed to determine the effects of Gyps on the hepatic FXR gene and protein expression levels in the different groups. As shown in Figure 5, hepatic FXR mRNA and protein levels were significantly decreased in the HFD group compared to the ND group (*p* < 0.01), while Gyps or OCA treatment significantly elevated hepatic FXR mRNA and protein expression in the HFD + Gyps or HFD + OCA groups vs. the HFD group (*p* < 0.01; Figure 5). There was no significant difference in hepatic FXR mRNA and protein expression levels between the HFD + Gyps and HFD + OCA groups (*p* > 0.05). In addition, hepatic FXR protein expression levels were significantly lower in the FXR KO ND group compared to the ND group (*p* < 0.01; Figure 5).

**FIGURE 6:**
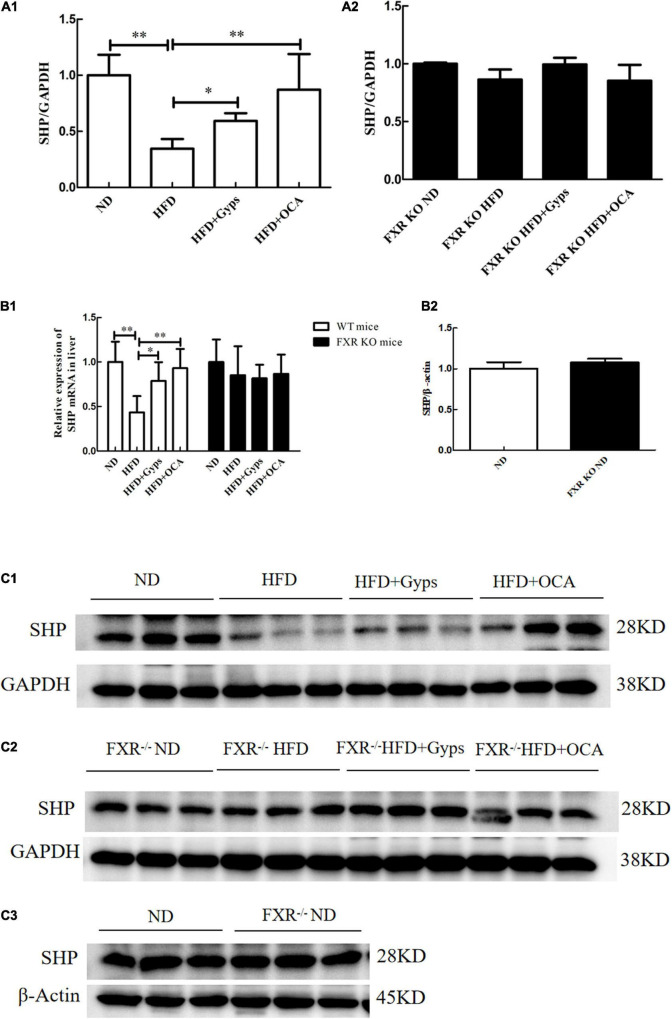
Effects of Gyps on hepatic SHP mRNA and protein levels in WT and FXR KO mice. **(A1)** Quantification of hepatic SHP protein levels in different groups of WT mice. **(A2)** Quantification of hepatic SHP protein levels in different groups in FXR KO mice. **(B1)** Relative expression levels of SHP mRNA in different groups in WT and FXR KO mice. **(B2)** Quantification of hepatic SHP protein levels in the ND and FXR KO ND groups. **(C1)** WB analysis of hepatic SHP protein expression in different groups of WT mice. **(C2)** WB analysis of hepatic SHP protein expression in different groups of FXR KO mice. **(C3)** WB analysis of hepatic SHP protein expression in ND and FXR KO ND groups. FXR, farnesoid X receptor; SHP, small heterodimer partner; ND, normal diet; HFD, high-fat diet; Gyps, gypenosides; WT, wild-type; KO, knockout; WB, western blot. *p < 0.05, ^**^p < 0.01.

In parallel, the effects of Gyps on hepatic SHP, a validated target gene of FXR, were examined. As illustrated in Figure 6, hepatic SHP mRNA and protein levels were significantly decreased in the HFD group compared to the ND group (*p* < 0.01), and treatment with Gyps led to a significant elevation of hepatic SHP mRNA and protein expression levels (*p* < 0.05) in WT mice. However, there were no significant differences in hepatic SHP mRNA and protein expression levels between the HFD + Gyps and HFD groups in FXR KO mice (*p* > 0.05). In addition, there was no significant difference in SHP mRNA and protein expression levels between ND and FXR KO ND groups (*p* > 0.05).

### Gyps down-regulated hepatic sterol-regulatory element binding protein 1, stearoyl-CoA desaturase 1, and fatty acid synthetase, and the effects were farnesoid X receptor-dependent

**FIGURE 7:**
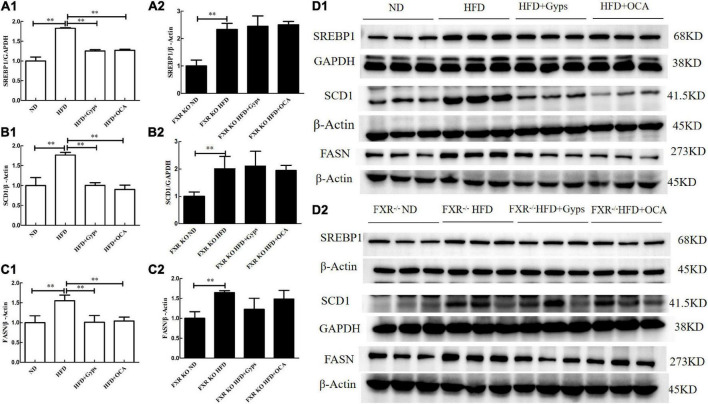
Effects of Gyps on hepatic SREBP1, SCD1, and FASN protein levels in WT and FXR KO mice. **(A1,A2)** Quantification of hepatic SREBP1 protein levels in the different groups of WT and FXR KO mice. **(B1,B2)** Quantification of hepatic SCD1 protein levels in different groups of WT and FXR KO mice. **(C1,C2)** Quantification of hepatic FASN protein levels in different groups of WT and FXR KO mice. **(D1,D2)** WB analysis of hepatic SREBP1, SCD1, and FASN protein expression in different groups of WT and FXR KO mice. SREBP1, sterol-regulatory element binding protein 1; SCD1, stearoyl-CoA desaturase 1; FASN, fatty acid synthetase; ND, normal diet; HFD, high-fat diet; Gyps, gypenosides; WT, wild-type; KO, knockout; WB, western blot ^**^p < 0.01.

**FIGURE 8:**
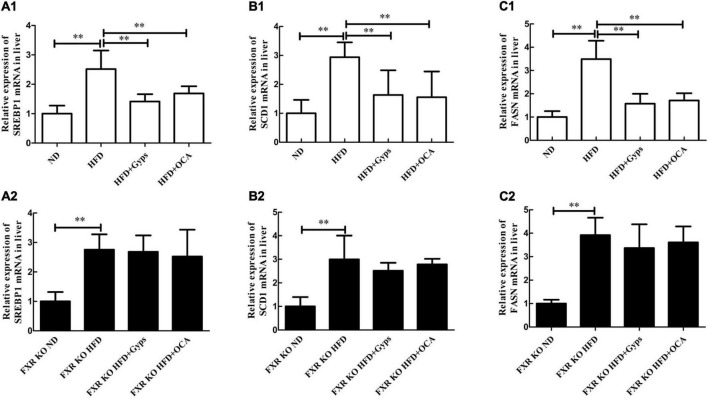
Effects of Gyps on hepatic SREBP1, SCD1, and FASN mRNA levels in WT and FXR KO mice. **(A1,A2)** Relative expression levels of SREBP1 mRNA in the different groups in WT and FXR KO mice. **(B1,B2)** Relative expression levels of SCD1 mRNA in different groups in WT and FXR KO mice. **(C1,C2)** Relative expression levels of FASN mRNA in different groups in WT and FXR KO mice. SREBP1, sterol-regulatory element binding protein 1; SCD1, stearoyl-CoA desaturase 1; FASN, fatty acid synthetase; ND, normal diet; HFD, high-fat diet; Gyps, gypenosides; WT, wild-type; KO, knockout; WB, western blot. ^**^p < 0.01.

Furthermore, the effects of Gyps on hepatic SREBP1, FASN, and SCD1 mRNA and protein expression levels in WT and FXR KO mice were determined. As presented in Figures 7, 8, the mRNA and protein expression levels of hepatic SREBP1, FASN, and SCD1 were greater in the HFD group vs. the ND group (*p* < 0.01) but were significantly lower in the HFD + Gyps group vs. the HFD group (*p* < 0.01) in WT mice. There were no significant differences in hepatic SREBP1, SCD1, and FASN mRNA and protein expression levels between the HFD + Gyps and HFD + OCA groups (*p* > 0.05).

Similar WB analysis was performed to examine effects of Gyps on hepatic SREBP1, FASN, and SCD1 mRNA and protein expression levels in FXR KO mice. Notably, there were no significant differences in hepatic SREBP1, SCD1, and FASN between the HFD + Gyps and HFD groups in FXR KO mice (*p* > 0.05; Figures 7, 8). These data indicated that the Gyps-mediated down-regulation of hepatic SREBP1, SCD1, and FASN expression was dependent on FXR.

### Gyps up-regulated hepatic carnitine palmitoyltransferase 1 and lipoprotein lipase, and the effects were dependent on farnesoid X receptor

**FIGURE 9:**
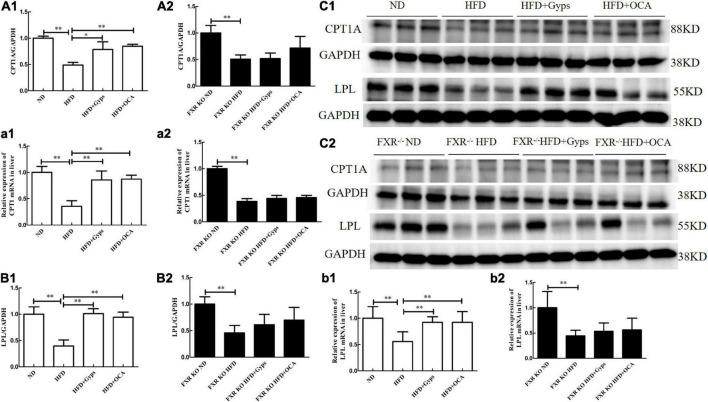
Effects of Gyps on hepatic CPT1A and LPL mRNA and protein expression in WT and FXR KO mice. **(A1,A2)** Quantification of hepatic CPT1A protein levels in different groups of WT and FXR KO mice. **(a1,a2)** Relative expression levels of CPT1 mRNA in different groups in WT and FXR KO mice. **(B1,B2)** Quantification of hepatic LPL protein levels in different groups of WT and FXR KO mice. **(b1,b2)** Relative expression levels of LPL mRNA in different groups in WT and FXR KO mice. **(C1,C2)** WB analysis of hepatic CPT1A and LPL protein expression in different groups of WT and FXR KO mice. CPT1A, carnitine palmitoyltransferase 1A; LPL, lipoprotein lipase; ND, normal diet; HFD, high-fat diet; Gyps, gypenosides; WT, wild-type; KO, knockout; WB, western blot. *p < 0.05, ^**^p < 0.01.

Lastly, the effects of Gyps on hepatic CPT1 and LPL mRNA and protein expression levels in WT and FXR KO mice were investigated. RT-PCR and WB analyses revealed that the mRNA and protein expression of hepatic CPT1A and LPL were significantly lower in the HFD group vs. the ND group (*p* < 0.01), while Gyps treatment mediated the up-regulation of CPT1A and LPL mRNA and protein expression in WT mice (*p* < 0.01 or *p* < 0.05; Figure 9). There were no significant differences in hepatic CPT1A and LPL mRNA and protein expression levels between the HFD + Gyps and HFD + OCA groups. As hypothesized, the RT-PCR and WB analyses in FXR KO mice showed no significant differences in hepatic CPT1A and LPL mRNA and protein expression levels between the HFD + Gyps and HFD groups (*p* > 0.05; Figure 9). These findings demonstrated that the Gyps-induced up-regulation of hepatic CPT1A and LPL was FXR-dependent.

## Discussion

**FIGURE 10:**
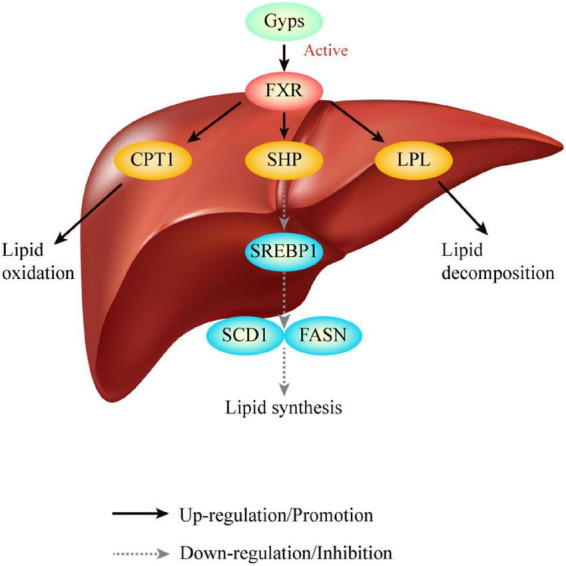
Graphical abstract. Gyps treatment significantly increased hepatic expression of FXR and its target SHP, and led to the up-regulation of CPT1 and LPL, and down-regulation of SREBP1, FASN and SCD1 protein levels in WT mice but not FXR KO mice. Ultimately, Gyps improves lipid metabolism in a mouse model of NASH through the activation of FXR. Gyps, gypenosides; FXR, farnesoid X receptor; SHP, small heterodimer partner; SREBP1, sterol-regulatory element binding protein 1; SCD1, stearoyl-CoA desaturase 1; FASN, fatty acid synthetase; CPT1A, carnitine palmitoyltransferase 1A; LPL, lipoprotein lipase.

This study built upon our previous findings that Gyps effectively inhibited hepatic lipid deposition involving the up-regulation of FXR in a mouse model of NASH. However, it remained unclear whether Gyps exerted its effects to improve lipid metabolism through the up- regulation of the FXR signaling pathway. The present study using WT and FXR KO mice has the following major novel findings: (1) Gyps treatment significantly improved liver histopathological abnormalities in HFD-induced NASH; (2) Gyps treatment decreased TG content in the liver and biochemical parameters associated with the dysregulation of lipid and glucose metabolism; (3) Gyps treatment significantly increased hepatic expression of FXR and its target SHP; and (4) Gyps treatment led to up-regulation of CPT1 and LPL, and down-regulation of SREBP1, FASN and SCD1 protein levels in WT mice but not FXR KO mice (Figure 10). Collectively, these data revealed a direct role of FXR in the Gyps-mediated effects in a mouse model of HFD-induced NSAH.

NAFLD represents a spectrum of liver injury conditions (e.g., simple steatosis, NASH), and is considered a liver manifestation of metabolic syndrome (2). Although the exact pathogenesis of NAFLD remains largely unknown, impaired hepatic lipid metabolism has been shown to be a key pathological mechanism (16). In addition, abnormal lipid metabolism is an important factor for the development of cardiovascular disease (CVD) in NAFLD patients and plays an important role in the progression of NAFLD to CVD (17). Under normal circumstances, hepatic lipid metabolism (lipid uptake, lipid synthesis, lipid transport, and fatty acid oxidation) remains in balance to maintain lipid homeostasis, but disruption of one or multiple pathways can lead to impaired hepatic lipid metabolism, resulting in excessive fat deposition and subsequently triggering NAFLD (18). Improving lipid metabolism disorders and restoring the balance and homeostasis of lipid metabolism have been shown to be effective in treating NAFLD in mice (19).

FXR is an important member of the nuclear receptor superfamily. It is primarily expressed in the liver and ileum and plays a key regulatory role in both glucose and lipid metabolism. Increasing evidence suggests that the occurrence of NAFLD is closely related to FXR dysfunction, and FXR is an important therapeutic target in the development of treatments for NAFLD (20). It has been found that the FXR agonist cilofexor (formerly GS-9674) can significantly reduce hepatic steatosis in NASH patients, and therefore has potential regarding the treatment of NASH (21). Another FXR agonist, MET409, significantly reduced the fat content in the livers of NASH patients following a 12-week treatment (22). Further mechanistic research has shown that FXR can regulate key components of lipid metabolism, such as hepatic lipid uptake, lipid synthesis, free fatty acid (FFA) oxidation, and lipid efflux. FXR is strongly associated with lipid metabolism homeostasis and plays an important role in hepatic steatosis in NAFLD (23). SHP is an important target gene of FXR in the liver, where the activation of FXR can inhibit protein expression of SREBP-1 by inducing SHP expression (24, 25). SREBP1 is a pivotal regulator of lipid synthesis, and its down-regulation can lead to the reduced expression of key genes involved in lipid synthesis, such as SCD1 and FASN (26, 27), thereby further impeding hepatic lipid synthesis. Additionally, the activation of FXR can also stimulate CPT1 and LPL expression, thereby promoting fatty acid oxidation and TG hydrolysis (28, 29). Aside from its function of regulating hepatic lipid metabolism, the activation of intestinal FXR can also promote the expression of fibroblast growth factor 15 (FGF15), which in turn decreases the expression of hepatic lipid synthesis-related genes such as SREBP1 and FASN, thereby inhibiting hepatic lipid synthesis and maintaining hepatic lipid homeostasis (30).

In this study, a 14-week HFD was used to induce NASH in WT and FXR KO mice. The H&E staining of liver sections revealed pathological characteristics of NASH, including steatosis with scattered inflammatory damage. The markedly increased hepatic TG content and serum ALT and AST activities, along with changes in serum lipids, suggests the successful establishment of HFD-induced NASH in mice. In addition, a 4-week treatment with Gyps was found to significantly improve pathological liver changes in HFD-induced NASH, significantly reduce liver TG content, and restore lipid homeostasis. These findings were consistent with our previous studies (12, 13). Notably, in FXR KO mice, the pharmacological effects of Gyps in HFD-induced NASH were significantly abrogated by the depletion of the *FXR* gene, suggesting a direct role of FXR in the Gyps-mediated effects. Therefore, FXR activation is an important mechanism underlying the therapeutic effects of Gyps on NASH. In this study, we found that there was no significant difference in serum HDL-C between all experimental groups, which was consistent with our previous study.

It merits attention in the current study that in WT mice, Gyps upregulated the hepatic protein expression of FXR and SHP, and thereby downregulated the expression of proteins involved in lipid synthesis, such as SREBP1, SCD1 and FASN, and upregulated the expression of proteins involved in lipid oxidation and hydrolysis, such as CPT1A and LPL. These results suggest that Gyps can activate FXR, and in turn induce the expression of SHP to achieve the pharmacological effect of regulating hepatic lipid metabolism, which supports the results of our previous study (12). Importantly, the Gyps-induced upregulation of SHP protein expression, along with the effects of Gyps on the protein expression of SREBP1, FASN, SCD1, CPT1A, and LPL, were abrogated in FXR KO mice. Based on the findings in WT and FXR KO mice, it was hypothesized that Gyps exerts its effects on hepatic lipid metabolism by directly activating FXR. Given that SHP is an important target gene of FXR in the liver and that SHP is strongly associated with lipid metabolism, it was hypothesized that the FXR-induced up-regulation of SHP participated in the pharmacological effect of Gyps on HFD-induced NASH in mice. It was found, however, that there was no significant difference in SHP mRNA and protein expression levels between the control ND and FXR KO ND groups. At present, we are unable to clearly explain these unexpected findings, since the regulatory mechanisms of a single target protein in the signaling network are very complex. However, based on several previous studies, there are some possible explanations. Firstly, in addition to FXR, other genes such as NF-E2-related factor 2 and fibroblast growth factor (FGF) 19 can also regulate SHP expression. Therefore, SHP can still be expressed in the absence of FXR (31, 32). Secondly, in addition to SHP, FGF15 is a critical target protein of FXR in the intestine. FXR can also regulate some target genes involved in lipid and bile acid metabolism, such as carnitine palmitoyltransferase 1, LPL, etc. Thus, FXR exerts its biological functions through several target genes (23). Still, the specific mechanism requires further investigation.

Previous studies have found that FXR is strongly associated with inflammatory damage in NASH (33) and that the activation of FXR can effectively improve liver inflammation in NASH (34, 35). The current results showed that Gyps improved liver inflammation and reduced serum ALT and AST activities while activating FXR in a mouse model of NASH. The Gyps-mediated pharmacological effects were abrogated in FXR KO mice, indicating a direct role for FXR in the Gyps-associated reduction of liver inflammation in a murine model of HFD-induced NASH, and that this effect was achieved through FXR activation. Aside from the dysregulation of lipid metabolism in NASH, insulin resistance and the disruption of glucose metabolism both play important roles in the pathogenesis of NAFLD. Studies have found that glucose levels and concentrations of insulin in NAFLD patients are significantly increased and that insulin sensitivity is closely related to hepatic lipid metabolism. Insulin resistance can promote hepatic lipid synthesis (36). HOMA-IR has a strong correlation with hepatic inflammation in NAFLD patients (37), and can estimate the progression of NAFLD, including the transformation of steatosis into steatohepatitis and the transformation of steatohepatitis into fibrosis and cirrhosis (38). Improving insulin sensitivity or reversing insulin resistance is a potential treatment strategy for NAFLD (39). It has been noted that FXR is closely associated with insulin sensitivity. FXR activation can regulate glucose-induced insulin transcription and secretion through genomic and non-genomic activities and can delay the occurrence of diabetes and hyperglycemia (40). It has been found that chenodeoxycholic acid (CDCA), an endogenous FXR agonist, can regulate the expression of adipokines to improve insulin resistance (41). Intestinal FXR agonists can promote adipose tissue browning and reduce obesity and insulin resistance, suggesting that FXR activation could be a therapeutic strategy for treating obesity and metabolic syndrome (42). The FXR agonist obeticholic acid (OCA) can normalize insulin sensitivity in visceral preadipocytes as well as improve liver function and adipose tissue function in rabbits with metabolic syndrome (43). In the present study, FINS, FBG, and HOMA-IR were significantly increased in HFD-induced NASH in WT mice, suggesting the presence of insulin resistance during HFD-induced NASH. Gyps can significantly reduce FINS, FBG, and HOMA-IR in HFD-induced NASH, suggesting that Gyps can increase insulin sensitivity and improve insulin resistance. After deletion of the *FXR* gene, the Gyps-mediated reductions of FINS, FBG, and HOMA-IR were significantly abrogated in FXR KO mice, suggesting that the effects of Gyps were FXR-dependent, and Gyps improved insulin resistance through FXR activation.

In summary, our results showed that Gyps activated FXR to regulate lipid metabolism, inhibit hepatic lipid deposition, improve insulin resistance, and reduce inflammatory liver damage in an HFD-induced model of NASH in mice. Gyps had significant therapeutic effects on NASH, similar to OCA, and thus holds potential as a novel therapeutic approach for the treatment of NASH. As such, this study improved our understanding of mechanisms underlying the therapeutic effects of Gyps on NASH and lays the foundation for a novel treatment in patients with NASH. Future studies will be required to gain insight into the mechanism whereby Gyps activates FXR in NASH.

## Data availability statement

The raw data supporting the conclusions of this article will be made available by the authors, without undue reservation.

## Ethics statement

The animal study was reviewed and approved by Experimental Animal Ethics Committee of Shanghai University of Traditional Chinese Medicine.

## Author contributions

HSL conceived and designed the study and was primarily responsible for final content. HSL, XX, and YFX performed experiments and collected the data. HSL, XX, and HLL analyzed and interpreted the data. HSL and YFX wrote the original manuscript. HSL revised the manuscript. All authors contributed substantially to the preparation of the manuscript and approved the final version and agreed to be accountable for all aspects of work ensuring integrity and accuracy.

## Abbreviations

ALT: alanine aminotransferase
AST: aspartate aminotransferase
CPT1: carnitine palmitoyl transferase 1
CVD: cardiovascular disease
FASN: fatty acid synthetase
FBG: fasting blood glucose
FFA: free fatty acid
FGF15: fibroblast growth factor 15
FINS: fasting insulin
FXR: farnesoid X receptor
Gyps: gypenosides
HFD: high-fat diet
HOMA-IR: Homeostatic Model Assessment for Insulin Resistance
KO: knockout
LPL: lipoprotein lipase
NAFLD: non-alcoholic fatty liver disease
NAS: NAFLD activity score
NASH: non-alcoholic steatohepatitis
ND: normal diet
OCA: obeticholic acid
SCD1: stearyl coenzyme A desaturation enzyme 1
SHP: small heterodimer partner
SREBP1: sterol-regulatory element-binding protein 1
WB: western blot
WT: wild-type.
